# Bridging the implementation gap: a qualitative study of barriers to integrating psychological care into rheumatoid arthritis services in Iran

**DOI:** 10.1186/s12913-026-15165-y

**Published:** 2026-07-20

**Authors:** Masoomeh Mayeli

**Affiliations:** Faculty of Social Sciences, Semnan University, Semnan, Iran

**Keywords:** Rheumatoid arthritis, Psychological care, Implementation science, Qualitative research, Health services research, Iran, CFIR

## Abstract

**Background:**

Despite robust evidence and explicit guideline recommendations, psychological care is rarely integrated into routine Rheumatoid Arthritis (RA) services in Iran. This study explored the multilevel barriers to this implementation gap.

**Methods:**

A qualitative study guided by the Consolidated Framework for Implementation Research (CFIR) was conducted. Semi-structured interviews were held with 34 stakeholders (12 patients, 8 rheumatologists, 7 nurses, 4 psychologists, 3 hospital managers) in Iranian rheumatology clinics. Data were analyzed using directed content analysis.

**Results:**

Barriers interacted across CFIR domains, forming an interpretive model of a reinforcing cycle. Financial exclusion (Outer Setting) reinforced biomedical clinic culture (Inner Setting), which diminished professional self-efficacy (Characteristics of Individuals) and nullified implementation processes. Stigma and lack of structured pathways were key themes.

**Conclusion:**

The integration gap reflects systemic misalignment, not a lack of evidence. Closing it requires coordinated policy, organizational, and educational reforms, not isolated interventions.

**Supplementary Information:**

The online version contains supplementary material available at https://doi.org/10.1186/s12913-026-15165-y.

## Introduction

Rheumatoid Arthritis (RA) is a chronic illness with profound biopsychosocial consequences. Extensive international evidence demonstrates that psychological factors such as depression, anxiety, pain catastrophizing, and fatigue play a critical role in shaping key clinical outcomes in RA, including pain intensity, treatment adherence, functional disability, disease activity, and overall quality of life [20, 24]. Accordingly, global clinical guidelines increasingly recognize evidence-based psychological interventions—such as cognitive-behavioral therapy and mindfulness-based approaches—as integral components of comprehensive RA care [24].

The biopsychosocial model of illness conceptualizes disease activity, psychological distress, and social functioning as dynamically interacting processes rather than independent domains [10, 17]. A substantial body of epidemiological evidence indicates that individuals with RA experience markedly elevated rates of psychological distress compared to the general population. Meta-analytic findings estimate the prevalence of depression in RA to range from approximately 16% to over 40%, while anxiety symptoms may affect a majority of patients depending on disease severity and assessment approach [18, 20, 4]. These rates far exceed population norms and highlight psychological distress as a core component of the RA disease burden. Importantly, depression and anxiety in RA are consistently associated with poorer clinical and functional outcomes, including exacerbated pain perception, increased fatigue [16], impaired physical functioning, and reduced adherence to disease-modifying antirheumatic drugs [7, 22]. In addition to affective symptoms, maladaptive cognitive processes—particularly pain catastrophizing—play a critical role in shaping patients’ experiences of RA. Pain catastrophizing, characterized by rumination, magnification, and feelings of helplessness, has been identified as a key mediator between disease activity and adverse outcomes such as heightened pain, emotional distress, and disability, independent of depressive symptoms [9]. Recent syntheses confirm the pervasive influence of catastrophizing across rheumatic diseases, with emerging RA-specific evidence linking higher levels of rumination and helplessness to increased disease activity and symptom burden [33, 34].

Against this backdrop, systematic reviews and meta-analyses consistently demonstrate that psychological interventions such as cognitive-behavioral therapy (CBT), mindfulness-based approaches, and coping skills training produce small-to-moderate but clinically meaningful improvements in pain, depressive symptoms, fatigue, and functional disability among patients with RA [2, 28, 31, 32, 20]. More recent studies further suggest that digitally delivered CBT-based interventions may enhance scalability while yielding significant improvements in mental health–related quality of life, anxiety, depression, and fatigue [4]. Consequently, international clinical guidelines now explicitly endorse psychological and behavioral interventions as integral elements of multimodal RA care.

Despite this strong evidence base and normative consensus, the translation of integrated psychological care into routine rheumatology practice remains limited across many health systems. Implementation science consistently demonstrates that evidence-based interventions often fail to be adopted, scaled, or sustained in routine healthcare settings—a phenomenon commonly described as the implementation gap [6, 21]. In rheumatology, this gap is evident in persistently low rates of referral to psychological services despite high levels of patient need, as well as in challenges implementing even core biomedical strategies [19, 11]. Early work by Sharpe et al. (2008) demonstrated that while CBT can significantly improve outcomes in RA, its integration into routine care is highly contingent on contextual factors such as leadership support, funding structures, and interprofessional collaboration [27]. Across healthcare systems, commonly reported barriers to integrating psychological care include limited consultation time, inadequate reimbursement mechanisms, fragmented care pathways, insufficient mental health training among physicians, and persistent stigma surrounding mental illness [3, 30]. Recent qualitative studies employing implementation frameworks further highlight obstacles at multiple levels, including healthcare professionals’ knowledge and attitudes, organizational capacity and resources, and weaknesses in interprofessional communication [5].

In low- and middle-income countries (LMICs), including Iran, this implementation gap is likely to be deeper and shaped by a constellation of structural, organizational, cultural, and policy-related constraints [15]. Within the Iranian health system, although formal policies exist to integrate mental health services into primary healthcare—dating back to their incorporation into the national Primary Health Care (PHC) network in 1988—multiple evaluations indicate that this integration remains fragmented in practice, particularly in urban areas and in specialized medical fields such as rheumatology [29, 8]. General barriers to mental healthcare utilization in Iran are well documented, including limited insurance coverage, high out-of-pocket costs, stigma associated with mental illness, shortages of specialized mental health professionals, unfavorable working conditions, gaps in workforce training, and limited intersectoral collaboration [29, 1, 8, 26]. Within the specific context of RA, Iranian research has largely focused on quantifying the prevalence of psychological comorbidity; a systematic review and meta-analysis estimated depression prevalence among Iranian RA patients to exceed 65%, highlighting an exceptionally high burden of unmet mental health needs [12, 13, 25]. However, little is known about how these systemic barriers intersect with the specific clinical, organizational, and professional dynamics of RA care. More importantly, the lived experiences and perspectives of key stakeholders—rheumatologists, nurses, psychologists, hospital managers, and patients—have remained largely unexplored. Notably absent from the Iranian literature are in-depth qualitative investigations that examine how organizational structures, professional roles, policy constraints, and patient experiences interact to shape the delivery—or absence—of psychological care within RA services.

To date, no study has systematically applied a comprehensive implementation framework, such as the Consolidated Framework for Implementation Research (CFIR), to qualitatively investigate the barriers to integrating psychological care into RA services in Iran from a multi-stakeholder perspective. Addressing this gap is essential for moving beyond problem description toward the development of feasible, contextually appropriate implementation strategies. The objective of this study is to identify and explain barriers to integrating psychological care into rheumatology services in Iran from the perspectives of patients, rheumatologists, nurses, and mental health providers, using the Consolidated Framework for Implementation Research (CFIR) as an organizing framework.

## Methods

### Study setting

The study was conducted within the Iranian healthcare system, focusing on both public and private rheumatology clinics and hospitals in urban settings (Tehran and Isfahan). Five clinical sites were included: three public tertiary rheumatology clinics affiliated with Tehran and Isfahan Universities of Medical Sciences, and two private rheumatology outpatient centers in Tehran. These settings were selected because they represent the primary points of care for RA patients and reflect the real-world organizational and policy contexts in which integration of psychological services would need to occur.

### Participants and sampling

A purposive and maximum variation sampling strategy was employed to ensure inclusion of diverse perspectives across the RA care continuum. A total of 34 participants were included:

- Patients diagnosed with RA (*n* = 12).
- Rheumatologists (*n* = 8).
- Nurses working in rheumatology units (*n* = 7).
- Clinical psychologists (*n* = 4).
- Hospital managers or health system policymakers (*n* = 3).

Inclusion criteria for professionals included a minimum of two years of experience in RA-related care or mental health services. Patients were eligible if they had a confirmed diagnosis of RA and had been receiving care for at least one year. Sampling continued until data saturation was achieved, defined as the point at which no new themes emerged from consecutive interviews.

### Data collection

Data were collected through semi-structured, in-depth individual interviews conducted between July and December 2025. Separate interview guides were developed for each stakeholder group and were informed by CFIR domains. The interview sequence began with broad, open-ended questions (e.g., “Tell me about your experience with psychological care for RA patients”), followed by CFIR-aligned probes only after spontaneous responses were elicited. This sequencing was designed to minimize confirmation bias and capture emergent themes before introducing framework-specific prompts.

Interviews explored experiences with psychological distress among RA patients, current care practices, perceived barriers and facilitators to integration, interprofessional collaboration, organizational readiness, and policy-level influences. All interviews were conducted in Persian, audio-recorded, and transcribed verbatim. Each interview lasted between 45 and 75 min.

### Data analysis

Data were analyzed using a directed qualitative content analysis approach guided by the Consolidated Framework for Implementation Research (CFIR) version 2022 [6]. The analysis proceeded through the following steps:

#### Step 1 – Familiarization

Two researchers independently read all transcripts to gain an overall understanding of the data.

#### Step 2 – Inductive coding (emergent themes)

Before applying CFIR constructs, both coders independently identified emergent themes using conventional content analysis. This step captured participant-driven concepts not anticipated by the framework.

#### Step 3 – Directed coding (CFIR mapping)

In a second pass, codes were mapped to CFIR constructs. The following CFIR domains and 12 specific constructs were applied: Intervention Characteristics (complexity, relative advantage), Outer Setting (external policy and incentives, patient needs and resources), Inner Setting (culture, implementation climate, readiness for implementation), Characteristics of Individuals (self-efficacy, knowledge and beliefs about the intervention), and Process (planning, champions, executing).

#### Step 4 – Operationalization of constructs

Evidence for each construct was defined as explicit statements, recurring patterns, or strong implicit references to that determinant. For example, any mention of “lack of insurance coverage for psychotherapy” was coded under Outer Setting – external policy and incentives.

#### Step 5 – Handling data that did not fit CFIR

Any data that could not be mapped to existing CFIR constructs were retained as inductive codes and reported separately as “emergent themes” in the findings. These were not forced into the framework.

The following CFIR constructs were not represented in the data or had minimal supporting evidence: Intervention Characteristics (design quality and packaging, cost), Inner Setting (available resources, access to knowledge and information), and Characteristics of Individuals (other personal attributes). These constructs were not forced into the analysis and are not discussed in the findings.

#### Step 6 – Cross-level mapping and cascade identification

To identify patterns of multilevel barriers, we examined the co-occurrence of codes across CFIR domains within the same participant and across stakeholder groups. A “cascade” or “recursive loop” was inferred when three or more participants from different stakeholder groups described a sequential chain of barriers (e.g., lack of insurance → biomedical culture → low self-efficacy → no implementation process). This cascade is presented as a conceptual model derived from participant accounts, not as a causal test.

#### Step 7 – Reliability and discrepancy resolution

Coding was independently performed by two researchers. Inter-coder agreement was calculated on the first six transcripts (kappa = 0.78). Discrepancies were resolved through discussion and consensus involving a third researcher.

### Trustworthiness

Trustworthiness was ensured through credibility, dependability, confirmability, and transferability [refer to Lincoln & Guba].

- **Credibility**: Prolonged engagement with the field (6 months), triangulation of data sources across multiple stakeholder groups, and member checking with five selected participants.
- **Dependability**: An audit trail documenting all analytic decisions, coding revisions, and team discussions was maintained.
- **Confirmability**: Reflexive memos were written throughout data collection and analysis to bracket the researcher’s assumptions.
- **Transferability**: Thick descriptions of the study context, participant characteristics, and settings are provided to allow readers to assess applicability to other contexts. However, findings are primarily transferable to urban academic and private rheumatology settings in Iran.

### Mitigation of confirmation bias (CFIR and interview probes)

Given that both the interview guide contained CFIR-aligned probes and the analysis used directed CFIR coding, the risk of confirmation bias was explicitly addressed through the following strategies:

- **Question sequencing**: Open-ended exploratory questions preceded all CFIR-specific probes.
- **Two-phase coding**: Inductive coding (without CFIR) was completed before directed CFIR mapping.
- **Emergent theme tracking**: A separate log of themes that arose spontaneously (without probes) was maintained and reported as a supplementary file.
- **Negative case analysis**: Two patients who had successfully accessed psychological care (via private payment) were analyzed separately to identify disconfirming evidence. Their accounts did not fit the dominant barrier model and are discussed in Supplementary File 2.

### Ethical considerations

Ethical approval for this study was obtained from the Ethics Committee of the University of Semnan. All procedures involving human participants were performed in accordance with the ethical standards of the institutional and/or national research committee and with the 1964 Helsinki Declaration and its later amendments. Written informed consent was obtained from all individual participants included in the study. Participants were assured of confidentiality and their right to withdraw at any time without consequences.

## Findings: a multilevel, interacting analysis of barriers using the cfir framework

Data analysis revealed a set of interrelated and mutually reinforcing barriers across all five CFIR domains. Rather than operating in isolation, these barriers were described by participants as forming a cascading configuration of structural, organizational, professional, and individual constraints that were consistently reported to hinder the integration of psychological care into routine RA services. The following findings are organized by CFIR domain. Emergent themes that arose spontaneously before CFIR probes are presented as reported; no themes were forced into the framework.

### Intervention characteristics: psychological care as complex, time-consuming, and non-core

Psychological interventions were widely perceived as incompatible with the dominant biomedical workflow of rheumatology clinics, reinforcing their marginalization as “non-essential” services.

#### Theme: Perceived complexity

Many medical professionals viewed these interventions as time-consuming and outside their core expertise. One rheumatologist noted:

*I have 15 minutes for a patient consultation. How can I address both joint inflammation and deep psychological issues in this time? This requires different expertise and time.”* (Rheumatologist, Male, 20 years of experience)

Participants described this perception as directly translating into non-referral behavior, as clinicians defaulted to what they defined as more straightforward and legitimate “core” biomedical tasks.

#### Theme: Lack of relative advantage

Several patients did not see the direct relevance of psychological interventions to their physical pain. A female patient expressed:

*The doctor gave me pills for my joint pain. How will talking about my stress help reduce the swelling in my knees?* (Patient, Female, 45 years old)

This perceived lack of relative advantage undermined patient demand, which participants felt further diminished clinicians’ motivation to prioritize and refer.

### Outer setting: policy, insurance, and stigma as system-level blockers

Financial and cultural barriers at the system level emerged as the most frequently cited and structurally decisive obstacles, shaping both organizational behavior and patient engagement.

#### Theme: Lack of insurance coverage

All groups unanimously cited this barrier as a foundational non-starter. A private clinic manager explained:

*Psychotherapy sessions are not covered by basic insurance. Patients must pay out of pocket*,* which is a major and often insurmountable barrier for most. We cannot build a sustainable service model on this foundation.* (Health Service Manager, Male)

#### Theme: Societal stigma

Fear of labeling, especially among older patients, was a powerful deterrent that shaped clinical conversations. A nurse observed:

*Many patients fear that if they are referred to a psychologist*,* others will think they are ‘crazy.’ We must first navigate this stigma before we can even discuss the clinical benefits.* (Rheumatology Nurse, Female, 10 years’ experience)

### Inner setting: biomedical dominance and organizational disconnection

The strong biomedical culture within clinics was not merely a professional orientation but was actively reproduced through time constraints, lack of formal referral systems, and the absence of interdisciplinary routines.

#### Theme: Dominant biological culture

The primary focus in most settings was exclusively on controlling lab parameters and pharmacotherapy, which actively crowded out psychosocial considerations. A clinical psychologist stated:

*The prevailing view is that psychology is a luxury add-on. The unspoken rule is: ‘First*,* control the inflammation*,* then we might talk about stress.’ This sequential thinking is the very barrier to integration.* (Clinical Psychologist, Female, 8 years of experience)

#### Theme: Absence of clear referral pathways

No formal protocols or pathways existed, making referral an ad hoc, effort-laden task. A social worker pointed out:

*There is no ‘warm handoff’ system or even a shared list of trusted psychologists. Every referral feels like reinventing the wheel*,* relying on personal favors rather than institutional process.* (Social Worker, Female)

### Characteristics of individuals: professional insecurity and uneven commitment

These individual-level barriers were not rooted in resistance alone but largely reflected structural deficiencies in education, training, and institutional support.

#### Theme: Low self-efficacy

Many rheumatologists and nurses expressed a lack of skill and confidence in addressing psychological issues, tracing it back to their training. A younger rheumatologist admitted:

*We received zero training in this during residency. I wouldn’t know how to start a brief conversation about mental health*,* let alone screen for it properly. I fear opening a Pandora’s box I can’t manage.* (Rheumatologist, Female, 5 years of experience)

#### Theme: Variable belief in effectiveness

While some professionals were strong believers in the biopsychosocial model, their commitment wavered without systemic support. As a result, individual motivation alone proved insufficient to overcome entrenched structural inertia.

### Process: the missing architecture of implementation

Across all centers, implementation failure was not due to a lack of theoretical agreement but rather to the absence of operational leadership, structured planning, and sustained coordination mechanisms.

#### Theme: Absence of an implementation champion

A critical missing element was a dedicated leader. A senior rheumatologist noted:

*Everyone nods in agreement that it’s important*,* but no one owns it. There is no champion to navigate the bureaucracy*,* secure resources*,* and hold the team accountable. Without that driver*,* it will always remain a ‘good idea’ for the future.* (Rheumatologist, Male, 25 years of experience)

#### Theme: Lack of participatory planning

The design of care pathways excluded key stakeholders, particularly patients and mental health professionals, ensuring plans were misaligned with on-the-ground realities and needs.

### Cross-domain interaction of barriers

Importantly, findings indicated that barriers across CFIR domains were not independent. A clear cascade of interaction was evident from participant accounts: Financial disincentives at the policy level (Outer Setting) legitimized and reinforced the exclusively biomedical orientation of clinics (Inner Setting). This organizational culture, in turn, fostered low self-efficacy and variable commitment among clinicians (Characteristics of Individuals), as they received neither training nor institutional signals to value this care. This confluence ultimately manifested in the complete absence of structured implementation processes (Process).

As interpreted by the research team, this multilevel interaction suggested a reinforcing cycle that systematically marginalized psychological care within routine RA services. This conceptual model—derived from participant accounts rather than causal testing—explains not only what the barriers are, but also how they dynamically interact to actively reproduce the implementation gap over time.

### Disconfirming cases

Two patients in the sample (both from private clinic settings) reported having successfully accessed psychological care through out-of-pocket payment. Their accounts did not fit the dominant barrier model described above. Neither patient described stigma as a deterrent, and both reported that their rheumatologist had initiated the referral spontaneously. While these cases are not representative of the broader sample, they suggest that in contexts where financial barriers are overcome and individual clinician motivation exists, integration may be possible even without systemic change.

## Discussion

### Principal findings and theoretical contribution

This study explored the multilevel barriers to integrating psychological care into routine Rheumatoid Arthritis (RA) services in Iran using the CFIR framework. The findings revealed that the implementation gap is not the result of a single dominant obstacle but rather emerges from a complex interaction of barriers across policy, organizational, professional, and individual levels. Financial disincentives and stigma at the outer setting level, the dominance of biomedical culture at the inner setting level, professional insecurity at the individual level, and the absence of structured leadership and planning at the process level collectively formed what participants described as a reinforcing cycle of implementation failure.

Theoretically, this study extends prior implementation research in chronic disease care, which has documented similar multilevel barriers in other contexts [5, 23], by demonstrating how biopsychosocial integration fails not due to a lack of clinical evidence, but due to systemic misalignment between financial structures, professional training, and organizational logics. While the CFIR has been widely applied in high-income countries to analyze implementation determinants [6, 21], this study provides a contextualized application in a middle-income health system. It builds upon policy analyses highlighting the decisive role of insurance structures in the Iranian context [8, 26] and moves beyond them by using qualitative inquiry to uncover the interacting, lived experiences of barriers across the care continuum—a critical perspective previously missing from the literature.

### Intervention characteristics: complexity and marginalization of psychological care

Participants widely perceived psychological interventions as complex, time-consuming, and peripheral to the “core” medical mission of rheumatology clinics. This perception was described as directly undermining their relative advantage and adoption. Similar patterns have been reported in RA care systems in Europe and North America [19].

However, in the Iranian context, this marginalization was amplified by severe time constraints, lack of interdisciplinary routines, and rigid biomedical workflows. Unlike high-income settings where task-shifting or embedded mental health professionals are increasingly implemented [4, 15], Iranian clinics lacked the structural mechanisms to redistribute psychosocial responsibilities [8]. This reinforces the notion that perceived complexity is not an intrinsic property of the intervention itself, but a product of organizational design.

### Outer setting: insurance, stigma, and structural disincentives

Consistent with previous Iranian studies on mental health service utilization, lack of insurance coverage emerged as the most decisive structural barrier [8, 26]. When psychotherapy is positioned as an out-of-pocket luxury rather than a reimbursable clinical necessity, both patients and providers rationally deprioritize it. This finding aligns with international implementation research showing that financial incentives are among the strongest predictors of sustained service adoption [6, 21].

Stigma further compounded this barrier by suppressing both patient demand and physician referrals. Unlike some Western systems where mental health promotion has partially normalized psychological care in chronic illness, in Iran, psychological referral remained strongly associated with social labeling [1]. This dual burden of economic and cultural deterrence created a powerful outer-setting blockade that overwhelmed individual-level motivation, a dynamic also observed in other settings where mental health is heavily stigmatized [30].

### Inner setting: biomedical dominance and organizational disconnection

The dominance of a biomedical culture within rheumatology clinics was not merely attitudinal but structurally embedded through time allocation, performance indicators, and documentation systems that prioritize laboratory and pharmacological outcomes over psychosocial functioning. Similar biomedical dominance has been documented in hospital cultures globally, often acting as a key barrier to integrating psychosocial care [14, 27]; however, this study shows how such dominance becomes particularly resistant to change when unsupported by referral infrastructure, interdisciplinary protocols, or integrated care pathways.

This structural inertia was evidenced by the near-universal reliance on informal personal networks for referrals, in the absence of any formal mechanisms. This practice effectively transforms access to an evidence-based component of care from an entitleable ‘right’ into a ‘privilege’ contingent upon individual social capital. This finding crucially reveals how organizational frailties can perpetuate health inequities by making standard care dependent on personal connections rather than institutional policy, a dynamic noted in other contexts where systemic support is lacking [26].

### Characteristics of individuals: professional insecurity rather than resistance

At the individual level, lack of self-efficacy among physicians and nurses stemmed primarily from educational and training deficiencies, rather than ideological opposition to psychology. Younger clinicians explicitly acknowledged inadequate preparation for screening, referral, or basic psychosocial communication. This contrasts with some Western contexts where resistance to psychological integration is often rooted in professional boundary protection.

Importantly, professionals who had greater exposure to the biopsychosocial model exhibited higher readiness for collaboration, suggesting that belief in effectiveness is a modifiable determinant of implementation, provided that supportive structures are present.

### Process: the missing architecture of implementation

Across all sites, implementation failure was driven not by lack of conceptual agreement, but by the absence of operational leadership, structured planning, and designated implementation champions. This finding closely mirrors international evidence indicating that successful integration depends less on clinical enthusiasm and more on governance, coordination roles, and accountability mechanisms.

In the absence of participatory planning, both patients and psychosocial professionals were excluded from pathway design, reinforcing top-down biomedical control and further weakening system ownership of psychological care.

### Cross-level dynamics: a conceptual model of a reinforcing cycle

One of the most important contributions of this study is the identification of a cross-level cascade from participant accounts: policy-level financial exclusion (outer setting) reinforced organizational biomedical priorities (inner setting), which diminished clinicians’ confidence and motivation (individual characteristics), and ultimately resulted in the absence of structured implementation processes (process domain). This recursive loop—presented here as a conceptual model derived from participants’ accounts rather than a causal test—continuously reproduces the marginalization of psychological care within RA services.

This finding supports emerging implementation science arguments that barriers should not be addressed in isolation, but through coordinated multilevel strategies.

### Implications for practice and policy in Iran

The findings suggest several actionable implications:

- At the policy level, inclusion of psychological services for RA within basic insurance packages is a prerequisite for any sustainable integration.
- At the organizational level, development of formal referral pathways, interdisciplinary case conferences, and embedded mental health roles can weaken biomedical dominance.
- At the professional level, targeted training in screening and brief psychosocial interventions can strengthen self-efficacy.
- At the process level, appointing local implementation champions and establishing participatory planning structures are essential for translating intention into practice.

Without simultaneous investment across these levels, isolated interventions are unlikely to disrupt the existing cycle of implementation failure.

### Strengths and limitations

**Strengths** A key strength of this study is its theory-driven design using CFIR, combined with the inclusion of diverse stakeholders across the RA care continuum. This allowed for a nuanced, multilevel understanding of implementation barriers.

**Limitations** Several limitations should be acknowledged.

First, **cross-language translation.** Interviews were conducted in Persian, and selected quotes were translated into English by two independent bilingual researchers. While reconciliation was performed, back-translation was not conducted. Therefore, some nuances of meaning—particularly for culturally embedded constructs such as stigma and psychological distress—may not have been fully preserved. Readers should interpret translated quotes with this consideration.

Second, **interview-guide priming.** The interview guide included probes explicitly aligned with CFIR constructs (e.g., insurance coverage, stigma, interprofessional communication). While we mitigated this risk through open-ended questioning before probes and two-phase coding (inductive before directed), the possibility of confirmation bias cannot be entirely excluded. Emergent themes that arose spontaneously are reported separately in Supplementary File 2.

Third, **analytic-team inconsistency.** Two coders independently analyzed the first six transcripts with acceptable inter-coder agreement (kappa = 0.78). However, the lead author performed the final analysis and integration of all transcripts. This introduces the possibility of individual analytic style influencing final theme consolidation. Future research should consider sustained dual coding throughout the entire dataset.

Fourth, **transferability and scope.** The findings are context-specific. All sites were located in urban areas (Tehran and Isfahan), and the sample was predominantly drawn from public tertiary clinics. Therefore, the results are primarily transferable to urban academic and private rheumatology settings in Iran. Transferability to rural areas, primary care, or other chronic diseases will require separate investigation.

Fifth, as with all qualitative research, interpretations are shaped by researcher reflexivity and participant disclosure. We attempted to bracket assumptions through reflexive memo-writing, but complete neutrality is not claimed.

## Conclusion

This qualitative study, employing the CFIR framework, indicates that the persistent gap in integrating psychological care into Rheumatoid Arthritis (RA) services in Iran reflects systemic misalignment rather than a lack of evidence. Based on participant accounts, we identified a reinforcing cycle in which policy-level financial exclusion reinforces biomedical organizational cultures, which in turn fuels professional insecurity and a complete absence of implementation leadership. Consequently, psychological care remains systematically marginalized as an optional adjunct rather than a fundamental pillar of biopsychosocial RA management.

The critical implication is that isolated clinical or educational initiatives are unlikely to succeed within this misaligned system. Closing this implementation gap requires a fundamental realignment of the system itself. This necessitates coordinated, multilevel action: policy reform to ensure insurance coverage, organizational redesign to establish formal interdisciplinary pathways, targeted professional development to build core competencies, and the appointment of implementation champions to drive and sustain the process. Only through such an integrated approach can evidence-based psychological care be translated into routine practice, ultimately improving the health and well-being of individuals living with RA in Iran.

## Supplementary Information

Below is the link to the electronic supplementary material.

Supplementary Material 1

## Data Availability

The qualitative datasets (interview transcripts) generated and analyzed during the current study are not publicly available due to their containing information that could compromise the privacy of research participants. Anonymized data may be available from the corresponding author on reasonable request.

## Abbreviations

CFIR: Consolidated Framework for Implementation Research
RA: Rheumatoid Arthritis
LMICs: Low- and Middle-Income Countries
CBT: Cognitive-Behavioral Therapy
