# Supplementary material for: Bridging the implementation gap: a qualitative study of barriers to integrating psychological care into rheumatoid arthritis services in Iran
Source: BMC Health Serv Res. 2026 Jul 20;26:1414. doi: 10.1186/s12913-026-15165-y (PMC13673759; doi:10.1186/s12913-026-15165-y)
Supplement: Supplementary file 1 [file 12913_2026_15165_MOESM1_ESM.docx]

**File 1**

**Semi-Structured Interview Guide (CFIR-Informed, English Version)**

**Study Title:
*Bridging the Implementation Gap: A CFIR-Guided Qualitative Study of Barriers to Integrating Psychological Care into Rheumatoid Arthritis Services in Iran***

**Note for Publication**

This semi-structured interview guide was developed specifically for the present study and was originally administered in Persian. The guide was informed by the Consolidated Framework for Implementation Research (CFIR) to explore determinants of implementation across its five domains. This document represents the translated English version of the interview guide and is provided to enhance methodological transparency. This interview guide has not been previously published.

**A. Rheumatologists and Nurses**

*(Primary CFIR Domains: Inner Setting; Characteristics of Individuals)*

1. Could you describe your current role and professional experience in managing patients with Rheumatoid Arthritis (RA)?
2. In your routine clinical practice, how do you typically address the psychological or emotional well-being of patients with RA?

**Probes:**

- Do you routinely ask patients about mood, stress, or coping? If yes, how do you usually approach this?
- If not, what are the main reasons (e.g., time constraints, lack of training, uncertainty about referral pathways)?
- Can you describe a recent situation in which a patient’s psychological distress was evident? How did you respond?

3. What is your perception of the role of psychologists or other mental health professionals within multidisciplinary RA care?

**Probes:**

- Have you ever referred a patient with RA to a psychologist or mental health professional? Please describe that process.
- What factors would make you more willing or able to make such referrals in the future?

**B. Clinical Psychologists and Psychiatrists**

*(Primary CFIR Domains: Intervention Characteristics; Process)*

1. Could you describe your professional experience, if any, with patients who have chronic physical illnesses such as RA?
2. From your perspective, what are the main barriers to providing psychological services to patients with RA within the current healthcare system?

**Probes:**

- How do factors such as insurance coverage, stigma, or interprofessional communication influence service provision?
- What challenges exist in collaborating with rheumatology clinics?

3. How suitable do you consider common psychological interventions (e.g., cognitive-behavioral therapy, mindfulness-based approaches) for patients with RA?

**Probes:**

- Are any adaptations needed to better address the specific needs of this patient group?
- What would an ideal referral and collaboration pathway with rheumatology services look like?

**C. Patients with Rheumatoid Arthritis**

*(Primary CFIR Domains: Outer Setting; Intervention Characteristics)*

1. Could you tell me about your experience of living with Rheumatoid Arthritis and how it affects your daily life?
2. How has living with RA affected your emotional well-being, mood, or stress levels?

**Probes:**

- Can you describe a specific situation when these emotional challenges were tough?
- Has any member of your healthcare team (e.g., rheumatologist or nurse) ever asked you about these emotional aspects? How did that conversation go?

3. What are your thoughts about receiving psychological or emotional support as part of your RA care?

**Probes:**

- What factors might make you reluctant to seek such support (e.g., cost, stigma, perceived lack of relevance)?
- What would help you feel more comfortable discussing emotional well-being with your healthcare providers?

**D. Health Service Managers and Policymakers**

*(Primary CFIR Domains: Outer Setting; Process)*

1. From an administrative or policy perspective, how are psychosocial services currently positioned within specialized care pathways such as rheumatology?
2. What are the primary policy, financial, or structural barriers to formally integrating psychological care into rheumatology services?

**Probes:**

- How do current reimbursement or insurance models support or hinder this integration?
- What systemic changes would be required to create sustainable funding for psychological care in RA services?

3. In your view, what key steps and which stakeholders would be necessary to develop and implement a structured, integrated care pathway for RA that includes psychological support?
